# Higher-order spin and charge dynamics in a quantum dot-lead hybrid system

**DOI:** 10.1038/s41598-017-12217-6

**Published:** 2017-09-22

**Authors:** Tomohiro Otsuka, Takashi Nakajima, Matthieu R. Delbecq, Shinichi Amaha, Jun Yoneda, Kenta Takeda, Giles Allison, Peter Stano, Akito Noiri, Takumi Ito, Daniel Loss, Arne Ludwig, Andreas D. Wieck, Seigo Tarucha

**Affiliations:** 1 Center for Emergent Matter Science, RIKEN, 2-1 Hirosawa, Wako, Saitama, 351-0198 Japan; 2 Department of Applied Physics, University of Tokyo, Bunkyo, Tokyo, 113-8656 Japan; 3 JST, PRESTO, 4-1-8 Honcho, Kawaguchi, Saitama, 332-0012 Japan; 4 Institute of Physics, Slovak Academy of Sciences, 845 11 Bratislava, Slovakia; 5 Department of Physics, University of Basel, Klingelbergstrasse 82, 4056 Basel, Switzerland; 6 Angewandte Festkörperphysik, Ruhr-Universität Bochum, D-44780 Bochum, Germany; 7 Quantum-Phase Electronics Center, University of Tokyo, Bunkyo, Tokyo, 113-8656 Japan; 8 Institute for Nano Quantum Information Electronics, University of Tokyo, 4-6-1 Komaba, Meguro, Tokyo, 153-8505 Japan

## Abstract

Understanding the dynamics of open quantum systems is important and challenging in basic physics and applications for quantum devices and quantum computing. Semiconductor quantum dots offer a good platform to explore the physics of open quantum systems because we can tune parameters including the coupling to the environment or leads. Here, we apply the fast single-shot measurement techniques from spin qubit experiments to explore the spin and charge dynamics due to tunnel coupling to a lead in a quantum dot-lead hybrid system. We experimentally observe both spin and charge time evolution via first- and second-order tunneling processes, and reveal the dynamics of the spin-flip through the intermediate state. These results enable and stimulate the exploration of spin dynamics in dot-lead hybrid systems, and may offer useful resources for spin manipulation and simulation of open quantum systems.

## Introduction

Electronic properties of quantum dots (QDs) have been widely studied to explore the solid-state physics of confined, interacting electrons^1–5^ and in addition consider various applications to quantum effect devices, quantum models, quantum information technologies and so on^6–8^. The QDs used are mostly isolated from their environment, including the leads, as much as possible to minimize dissipation and decoherence^9^. On the other hand, QDs coupled to their environment provide novel systems with the coupling electrically tunable. The environment can be tailored by applying bias voltages or using specific states such as ferromagnets^10^, superconductors^11^, quantum Hall states^12–14^, and others. This variability gives rise to attractive science like Fano interference^15–17^, RKKY interactions^18^, and the general physics of open and nonequilibrium systems. The higher order tunneling process in the open system also creates interesting phenomena like Kondo effects^19,20^. The higher order process occurs via transitions to and from the virtural intermediate states because of the time energy uncertainty principle. When such transitions happen, they can induce a spin change but no charge change between the initial and the final states. This is not the case for the first order tunneling process which accompanies a charge change with a spin change^21–23^. The difference between the two processes sounds obvious in quantum mechanics, and indeed has often been assumed to account for the exotic spin-related phenomena like the Kondo effect. However, most of the experiments have been performed using steady-state charge transport measurement^24,25^ and no direct measurement of time-dependent spin and charge changes has been demonstrated yet. Resolving the dynamics of higher order tunneling processes will therefore strengthen our understanding of the underlying physics of exotic spin phenomena.

In this work, we apply techniques of fast manipulation and readout of charge and spin states in a quantum dot coupled to the lead to directly reveal the time-dependent charge and spin change in the first and the second order processes induced by the dot-lead tunneling. Our experimental system is an electrostatically defined double quantum dot (DQD) in GaAs. One QD stores the target single-electron and another ancillary QD is used for spin initialization and readout. We measured time-dependent spin and charge changes and demonstrate the spin change with no charge change through the intermediate state in the second order tunneling process.

## Results

### Device and measurement scheme

**Figure 1:**
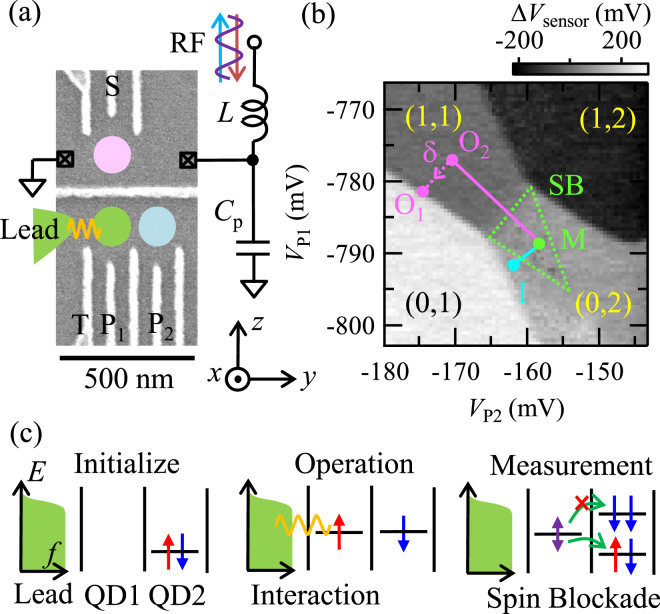
(**a**) Scanning electron micrograph of the device and the schematic of the measurement setup. A DQD is formed at the lower side, and the charge states are monitored by the charge sensor QD at the upper side. The charge sensor is connected to resonators formed by the inductor *L* and the stray capacitance *C*_p_ for RF reflectometry. The external magnetic field of 0.5 T is applied in plane along the *z* axis. (**b**) Δ*V*_s*ensor*_ as a function of *V*_P2_ and*V*_P1_. Charge states are identified by distinct levels in Δ*V*_s*ensor*_. The number of electrons in each QD is given as (*n*_1_, *n*_2_). The triangle shows the region of the spin blockade. The positions corresponding to steps of pulse sequences (O_*i*_, I, M) are indicated. (**c**) Schematic of the measurement scheme. The spin state is initialized to a (0, 2) singlet at I. Next, we move into O_*i*_ in (1, 1) where the spin couples to the lead. Finally, the spin state is measured using spin blockade at M.

Figure 1(a) shows a scanning electron micrograph of the device. By applying negative voltages on the gate electrodes, a DQD and a QD charge sensor^26^ are formed at the lower and upper sides, respectively. The left QD in the DQD couples to a lead, and the coupling strength is tuned by the voltage *V*_T_ applied on gate T. The QD charge sensor is connected to an RF resonator formed by the inductor *L* and the stray capacitance *C*_p_ for RF reflectometry^26–28^. The number of electrons in each QD (*n*_1_,*n*_2_) is monitored by the intensity of the reflected RF signal *V*_s*ensor*_.

The spin state is initialized using singlet formation in a single QD due to the tight confinement^29,30^. The charge state of single electrons in the dot can be detected in a fast and sensitive manner using the rf charge sensor. We combine the charge sensing with the effect of Pauli spin blockade^31^ to measure the spin change in the dot in a sufficiently short time scale.

Figure 1(b) shows the charge stability diagram of the DQD. The external magnetic field of 0.5 T is applied in plane along the *z* axis to create a large enough Zeeman splitting for the spin readout. We measure the sensor signal *V*_s*ensor*_ as a function of the plunger gate voltages of QD_2_ (*V*_P2_), and QD_1_ (*V*_P1_). We observe a change Δ*V*_s*ensor*_ each time the DQD charge configuration (*n*_1_, *n*_2_) changes. Depicted in Fig. 1(b), the values (*n*_1_, *n*_2_) are assigned by counting the number of charge transition lines from the fully depleted configuration (*n*_1_, *n*_2_) = (0,0). Around the charge state transition (1, 1) ↔ (0, 2), we observe a suppression of the (0,2) charge signal due to the Pauli spin blockade [in the region indicated by the triangle in Fig. 1(b)]. In this specific measurement of the stability diagram, unlike elsewhere, upon pulsing (0, 2) → (1, 1) we move through the singlet-triplet *T*_+_ anti-crossing very slowly (adiabatically), to induce a sizable triplet component of the (1, 1) state even at a zero interaction time. Pulsing quickly back (1, 1) → (0, 2) results in a Pauli blocked signal inside the denoted triangular area. This shows us where we can utilize the Pauli spin blockade to readout the spin state in the following measurements, probing the dot spin and charge tunneling-induced dynamics. The operation scheme to measure the effect of the lead on the spin is depicted in Fig. 1(c). We initialize the state to a (0, 2) singlet by waiting at the initialization point I denoted in Fig. 1(b) and return back to the point M. Next, we move to the operation point O_*i*_. In this step, the electron in QD_1_ interacts with the lead and the dot state might be changed by electron tunneling. The tunneling rate can be modified by tuning *V*_T_, which changes the tunnel coupling, and the position of O_*i*_, which changes the dot potential with respect to the Fermi energy of the lead (O_1_: close to a charge transition, O_2_: deep in the Coulomb blockade). In this detuned condition at O_*i*_, the single-electron dynamics in QD_1_ dominates over two-electron effects. At the next step, the spin state is measured using spin blockade by pulsing the dot to the point denoted by M. If the spin state has not changed, we observe the (0, 2) singlet again. If the spin state has changed, a polarized triplet component (*T*_±_) is measured as a blocked (1, 1) → (0, 2) charge transition. From the charge signal, we can therefore deduce the spin state.

### Measurement around a charge transition

**Figure 2:**
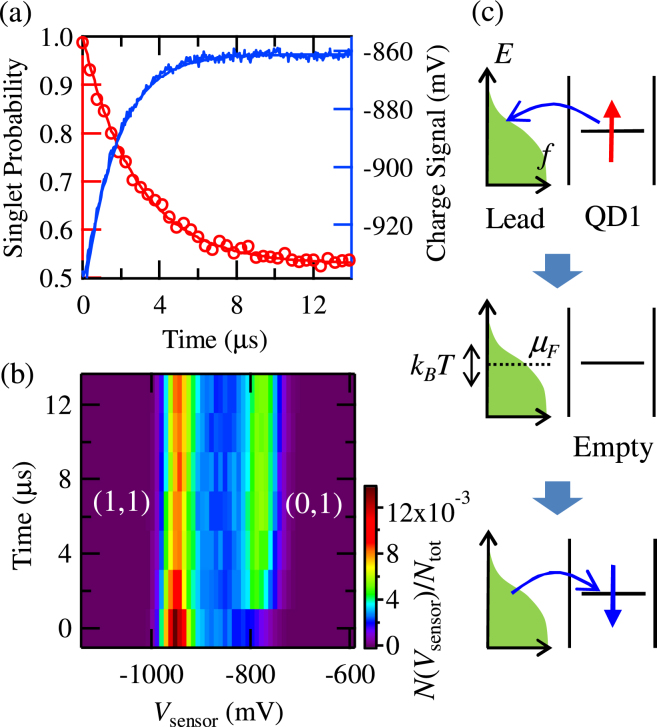
(**a**) Observed spin and charge signals (the singlet probability and the average of the sensor signal 〈*V*_s*ensor*_〉) as a function of the interaction time. Red circles show the spin signal (left axis). The blue trace shows the charge signal (right axis). The smooth lines are exponential fits resulting in the relaxation time of 3.0 *μ*s for the spin, and 1.8 *μ*s for the charge. (**b**) Statistics of the charge signal at the operation point. Histogram of observed values of the charge sensor *V*_s*ensor*_ (on the x axis), *N*(*V*_s*ensor*_)/*N*_t*ot*_ is plotted as a function of the interaction time (y axis). The two peaks, at *V*_s*ensor*_ = −960 mV, and −780 mV, correspond to the (1,1) and the (0,1) charge states, respectively. The weight of the (0,1) component increases with the longer interaction time. (**c**) Schematic of the spin relaxation by a first-order tunneling process. An electron escapes from the QD, and the QD becomes empty. Another electron comes in after that.

In this way, we first measure the spin relaxation using the operation point O_1_ close to a charge transition line, see Fig. 1(b), where the QD level is close to the Fermi level of the lead. The tunneling gate voltage is set to *V*_T_ = −660 mV. The red circles in Fig. 2(a) show the measured singlet probability as a function of the interaction time at O_1_. We average over 512 measurement cycles to produce a single data point. Initially at 1, the singlet probability decreases upon increasing the interaction time from zero. This decrease indicates that a triplet component is formed by the interaction with the lead. Fitting with an exponential reveals a relaxation time of 3.0 *μ*s. Note that this relaxation time is much smaller than the intrinsic spin relaxation time (several hundreds of *μ*s, ms)^29^.

Similarly to spin, we also measure the lifetime of charge in this configuration. In this measurement, we monitor the QD charge sensor while we are at O_1_. The blue trace in Fig. 2(a) shows *V*_s*ensor*_ over 16384 measurement cycles as a function of the interaction time. As seen there, 〈*V*_s*ensor*_〉 changes exponentially, with the fitted charge relaxation time of 1.8 *μ*s. To examine the charge relaxation in more detail, we plot in Fig. 2b histograms of the values of *V*_s*ensor*_ (the x axis) for a varying interaction time (the y axis). The two peaks along a horizontal cut correspond to the (1,1) and the (0,1) charge states, respectively. At zero interaction time, only the (1,1) state signal is present, while the (0,1) state appears for finite interaction times.

In this configuration, the mechanism of the relaxation for both spin and charge is a first-order tunneling process^32^. Namely, the electron tunnels out of the QD_1_ into the lead, after which the dot is refilled from the lead, and the initial information is lost. The spin and charge relaxations: the information loss of the spin demonstrated in Fig. 2(a) and of the charge in Fig. 2(a,b), happen simultaneously. We note that though the relaxation timescales are similar, they are not identical. The difference comes from a difference in the rate dependence on the Fermi occupation of the lead (see Supplementary Information).

### Measurement in Coulomb blockade

**Figure 3:**
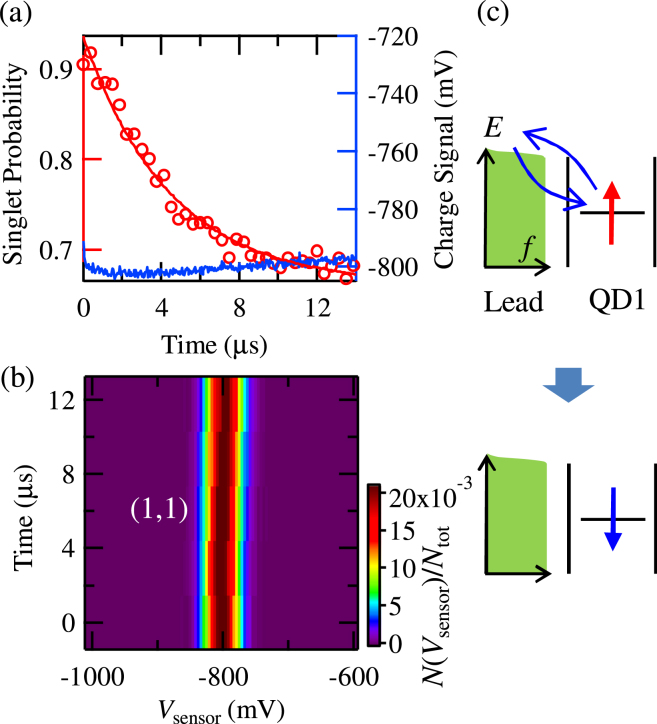
(**a**) The observed singlet probability and 〈*V*_s*ensor*_〉 as a function of the interaction time at O_2_ [see Fig. 1(b)]. Red circles show the spin signal (left axis). The blue trace shows the charge signal (right axis). The red smooth curve is an exponential fit resulting in the relaxation time of 4.5 *μ*s. The charge signal shows no relaxation. (**b**) Histogram of observed values of the charge sensor voltage *V*_s*ensor*_ (on the x axis), *N*(*V*_s*ensor*_)/*N*_t*ot*_, is plotted as a function of the interaction time (y axis). The peak corresponds to the (1,1) charge state. (**c**) Schematic of the spin relaxation by a second-order tunneling process. An electron of the QD_1_ is swapped with one in the lead in a single step. The spin state is changed even though the charge state is stable.

We now investigate the spin dynamics in a Coulomb blockaded dot. To this end, we repeat the previously described measurement using the operation point O_2_, deep in the (1,1) region, see Fig. 1(b). Here, the QD level is far below the Fermi level of the lead. To increase the speed of the lead-induced spin dynamics on the dot, we increase the dot-lead tunnel coupling by setting *V*_T_ =  −560 mV. As can be seen in Fig. 3(a), similarly to before, the spin state displays an exponential decay, with the relaxation time of 4.5 *μ*s. (The saturation value of the spin signal is slightly different from that in Fig. 2(a). This will be caused by an imperfection in the readout of the *T*_+_ state with increasing the dot-lead tunnel coupling.) However, now the charge signal barely changes, indicating that the charge state is not affected. (The slight change of the charge signal in Fig. 3(a) is caused by the distorted voltage pulses applied on P1 and P2. Due to a cross-talk between the plunger gates and the sensor, the pulse distortion slightly affects the observed charge signal.) This is confirmed by Fig. 3(b), where the histograms of the values of *V*_s*ensor*_ display a single peak corresponding to the (1,1) charge state. The spin therefore decays at a fixed QD charge configuration.

**Figure 4:**
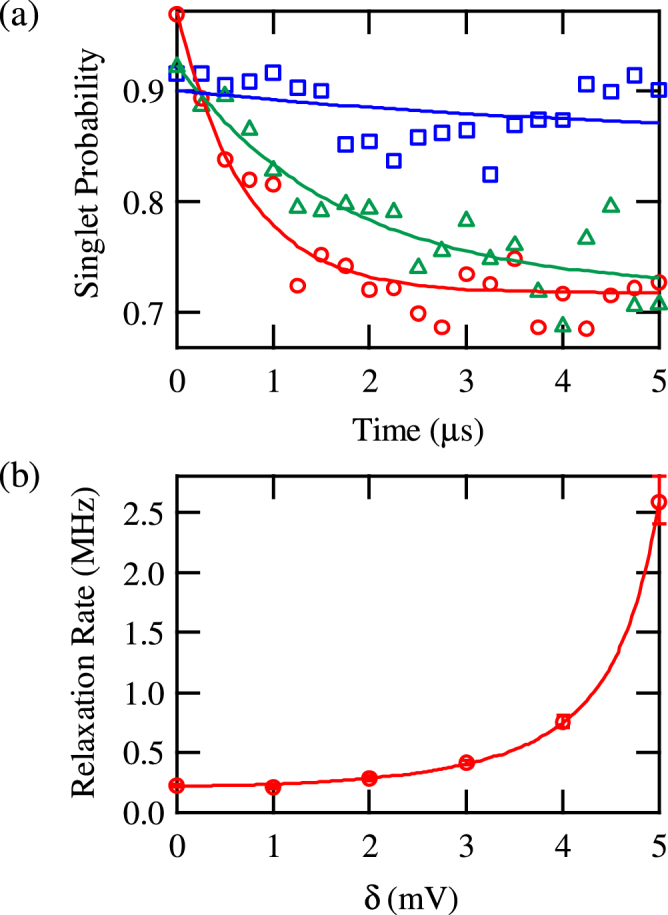
(**a**) The spin relaxation signal as a function of the interaction time at O_2_ for different values of the gate voltage applied on gate T, *V*_T_. Circles, triangles, and squares show the result at *V*_T_ = −560, −565, −570 mV, respectively. The lines are exponential fits. (**b**) The spin relaxation rate as a function of *δ*. Circles show the experimental data and the line shows a theoretical curve considering the second-order tunneling process.

We therefore interpret this as the observation of a spin relaxation induced by a second-order tunneling process^24,25^, where the electron in QD_1_ swaps with a random electron from the lead in a single step. Figure 4(a) shows the spin signal as we change the voltage applied on gate T, *V*_T_. Applying more negative voltage *V*_T_ prolongs the spin relaxation time, by decreasing the tunnel coupling to the lead, as 0.7, 1.7 and 5.0 *μ*s, for *V*_T_ = −560, −565, −570 mV, respectively. (We note that the relaxation time at *V*_T_ = −560 mV is different from the corresponding value of *V*_T_ given in Fig. 3(a) due to a shift of the QD conditions between experiments.) In addition to *V*_T_, we can tune the spin decay timescale by the plunger gate voltages. Figure 4(b) shows the spin relaxation rate as we change the operation point from O_2_ toward O_1_, parametrizing the displacement by the voltage *δ*. Upon increasing *δ* (moving towards the charge transition line), the spin relaxation rate is enhanced. The measured dependence is well fitted by an analytical expression for an inelastic cotunneling rate, giving $\propto {(\mathrm{1/(}\mu \mathrm{(2)}-{\mu }_{F})+\mathrm{1/(}{\mu }_{F}-\mu \mathrm{(1))})}^{2}$, with *μ*(*N*) and *μ*_*F*_ being the electrochemical potential at the dot with *N* electrons^29^ and the Fermi energy of the lead, respectively (see Supplemental Information for details). This demonstrates the two handles on the speed of the lead-induced dynamics of the QD spin.

## Discussion

To sum up the results observed in the Coulomb blockade regime, we state that the interaction with the lead influences only the dot spin and not its charge. The spin relaxation thus directly uncovers the second order tunneling processes. This interaction can be utilized for the spin initialization, measurement and manipulation if leads have special properties. We note that even though the timescale of the dot-lead interaction realized in this experiment was tuned to $\sim \mu$s, it is straightforward to enhance it by increasing the tunnel coupling, and/or utilizing the Kondo effect, which enhances the second-order tunneling at low temperatures.

In conclusion, we have measured spin dynamics in a QD-lead hybrid system. Close to a charge transition, we observe spin and charge relaxation signals corresponding to the first-order tunneling process. In the Coulomb blockade, we observe spin relaxation at a fixed charge configuration, corresponding to the second-order tunneling process. The demonstrated dot-lead spin exchange can be useful as a general resource for spin manipulations, and simulations of open systems under non-equilibrium conditions.

## Methods

The device was fabricated from a GaAs/AlGaAs heterostructure wafer with an electron sheet carrier density of 2.0 × 10^15^ m^−2^ and a mobility of 110 m^2^/Vs at 4.2 K, measured by Hall-effect in the van der Pauw geometry. The two-dimensional electron gas is formed 90 nm under the wafer surface. We patterned a mesa by wet-etching and formed Ti/Au Schottky surface gates by metal deposition, which appear white in Fig. 1(a). All measurements were conducted in a dilution fridge cryostat at a temperature of 13 mK.

The RF resonator for RF reflectometry is formed by the inductor *L* = 270 nH and the stray capacitance *C*_p_ = 1.06 pF. A change in the electrostatic environment around the sensing dot changes its conductance, which shifts the tank circuit resonance and modifies *V*_s*ensor*_ measured at *f*_r*es*_ = 297 MHz, the circuit resonance frequency.

## Electronic supplementary material

Supplemental Material
