# Supplementary material for: Higher-order spin and charge dynamics in a quantum dot-lead hybrid system
Source: Sci Rep. 2017 Sep 22;7:12201. doi: 10.1038/s41598-017-12217-6 (PMC5610234; doi:10.1038/s41598-017-12217-6)
Supplement: Supplementary file 1 — Supplemental Material [file 41598_2017_12217_MOESM1_ESM.pdf]

Supplemental Material to 'Higher-order spin and charge dynamics in a quantum dot-lead hybrid system'

Tomohiro Otsuka,[1,2,3,*] Takashi Nakajima,[1,2] Matthieu R. Delbecq,[1] Shinichi Amaha,[1] Jun Yoneda,[1,2] Kenta Takeda,[1] Giles Allison,[1] Peter Stano,[1,4] Akito Noiri,[1,2] Takumi Ito,[1,2] Daniel Loss,[1,5] Arne Ludwig,[6] Andreas D. Wieck,[6] and Seigo Tarucha[1,2,7,8,†]

[1]Center for Emergent Matter Science, RIKEN, 2-1 Hirosawa, Wako, Saitama 351-0198, Japan

[2]Department of Applied Physics, University of Tokyo, Bunkyo, Tokyo 113-8656, Japan

[3]JST, PRESTO, 4-1-8 Honcho, Kawaguchi, Saitama, 332-0012, Japan

[4]Institute of Physics, Slovak Academy of Sciences, 845 11 Bratislava, Slovakia

[5]Department of Physics, University of Basel, Klingelbergstrasse 82, 4056 Basel, Switzerland

[6]Angewandte Festkörperphysik, Ruhr-Universität Bochum, D-44780 Bochum, Germany

[7]Quantum-Phase Electronics Center, University of Tokyo, Bunkyo, Tokyo 113-8656, Japan

[8]Institute for Nano Quantum Information Electronics, University of Tokyo, 4-6-1 Komaba, Meguro, Tokyo 153-8505, Japan

(Dated: September 11, 2017)

CHARGE AND SPIN RELAXATION SIGNALS

We describe the dynamics of the charge and spin on the QD by considering the rate equation

$$\partial_t P_\sigma \;=\; -\Gamma_\sigma(1-f_\sigma)P_\sigma + \Gamma_\sigma f_\sigma P_{\rm e}, \tag{1}$$

for the probability P_σ that the dot is occupied by a single electron with spin $\sigma \in \{\uparrow,\downarrow\}$ (we alternatively use $\sigma = \pm 1$), where $P_{\rm e}$ is the probability that the dot is empty. We do not consider any other states, which gives the normalization condition $P_\uparrow + P_\downarrow + P_{\rm e} = 1$. Eq. (1) includes the process of an electron with a given spin leaving the dot into the lead where an empty state exists with the probability $(1-f_\sigma)$ and entering an empty dot from the lead state occupied with probability f_σ. This Fermi factor is given by $f_\sigma = f_{\rm FD}(\mu(1) - \sigma E_z/2)$, with $\mu(1) = eV_g + \epsilon_1$ being the energy cost to add an electron into the dot, which includes the electrostatic potential energy eV_g, and the orbital (quantization) energy ϵ_1. The Zeeman energy is $E_z = g\mu_B B$, and the Fermi-Dirac distribution

$$f_{\rm FD}(\epsilon) = \left\{\exp\left[\frac{\epsilon-\mu_F}{k_B T}\right]+1\right\}^{-1}, \tag{2}$$

depends on the temperature T, and the lead Fermi energy μ_F. Apart from the Fermi factors f_σ, the tunneling rates for hopping on and off the dot are identical, Γ_σ. We, however, allow for a spin dependence of the tunneling rate which has been found to be an appreciable effect (the asymmetry of the rates can be of the order of the rates themselves), most probably due to the exchange interaction in the lead [1–3].

To expose the spin and charge dynamics, we introduce new variables, the probability of charge occupation, $P_{\rm o} = P_\uparrow + P_\downarrow$ and the spin polarization, $s = P_\uparrow - P_\downarrow$, and new parameters, for the average, Γ, and the dimensionless asymmetry α, in the tunneling rates, by writing $\Gamma_\sigma = \Gamma(1+\sigma\alpha)$, and similarly for the Fermi factors, $f_\sigma = f + \sigma f_\delta$. Equation (1) can be now cast into the matrix form for the vector of unknowns, $v = (P_{\rm o}, s)^{\rm T}$, namely

$$\partial_t v = -M(v - v_0), \tag{3}$$

with the matrix defining the system propagator

$$M = \Gamma \begin{pmatrix} 1+f+f_\delta\alpha & -f_\delta + (1-f)\alpha \\ f_\delta + (1+f)\alpha & 1-f-f_\delta\alpha \end{pmatrix}, \tag{4}$$

and the steady state solution

$$v_0 = \frac{2}{1 - f^2 + f_\delta^2} \begin{pmatrix} f(1-f) - f_\delta^2 \\ f_\delta \end{pmatrix} . \tag{5}$$

The steady state is independent of the tunneling rates, and depends only on the lead Fermi factors for the two spins, as it should be, while the propagator matrix depends on all parameters of the problem. Even though it is straightforward to solve the problem in the most general case, it is useful to consider M for $\alpha = 0$ (spin independent tunneling rates), which gives

$$M = \Gamma \begin{pmatrix} 1+f & -f_\delta \\ f_\delta & 1-f \end{pmatrix} . \tag{6}$$

For a negligible difference of the Fermi function values for the two spins, $f_\delta \to 0$, the charge and spin decay to their steady state values independently, with the rates $\Gamma(1+f)$, and $\Gamma(1-f)$, respectively. The steady states are also markedly different in this limit, as $P_o(t=\infty) = 2f/(1+f)$ depends on the Fermi factors, while $s(t=\infty) = 0$ does not. This is then the reason for the difference in the decay time scales: while the charge decays towards the steady state with effectively the sum of the rates for leaving, $(1-f)\Gamma$, and entering, $2f\Gamma$, the dot, only the events of electrons leaving the dot can relax the spin polarization s. The spin and charge relaxation time scales will then be most different if $f \approx 1$, where the charge equilibrates much faster than the spin.

To demonstrate this difference, seen also experimentally, we plot the charge and spin signals in Fig. S1 (a) and (b), respectively. The parameters are set as $T = 200$ mK, $B = 0.5$ T, $g = -0.37$ [4]. The traces show the results with $f = 0.2, 0.4, 0.6, 0.8$ from the bottom to the top.

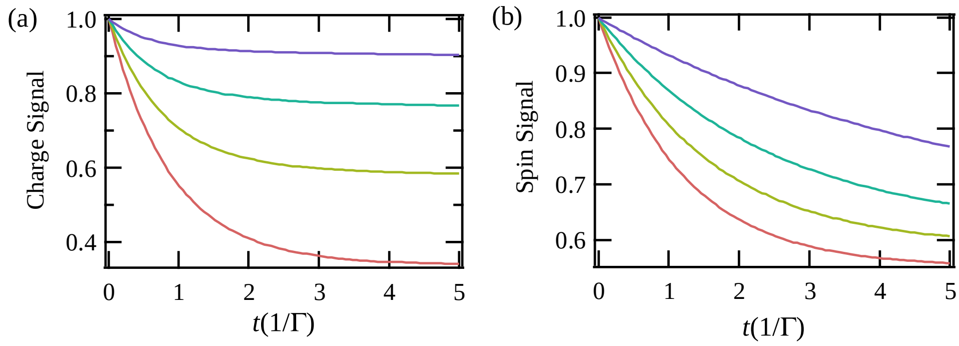

FIG. S1: (a) Calculated charge signal as a function of t. The traces show the results with $f = 0.2, 0.4, 0.6, 0.8$ from the bottom to the top. (b) Calculated spin signal as a function of t.

CO-TUNNELING RATE

To derive the formula for the spin relaxation by cotunneling, which was used in the main text to fit the data on Fig. 4(a), we consider the Hamiltonian of a QD coupled to a lead, $H = H_D + H_L + H_T$. Here the dot Hamiltonian is

$$H_D = \sum_{\alpha \in \{0,\sigma,S\}} \epsilon_\alpha |\alpha\rangle\langle\alpha|, \tag{7}$$

where the index α labels the states of the dot $|\alpha\rangle$ with energies ϵ_α, and $|0\rangle$ denotes an empty dot, $|\sigma\rangle = d_\sigma^\dagger|0\rangle$ a dot with a single electron with spin σ, and $|S\rangle = d_\uparrow^\dagger d_\downarrow^\dagger|0\rangle$ a dot with a two electron singlet state, and $d_\sigma^\dagger$ is the creation operator of a dot electron with spin σ. The lead is described by

$$H_L = \sum_{k\sigma} \epsilon_{k\sigma} c_{k\sigma}^\dagger c_{k\sigma}, \tag{8}$$

where k is a wave-vector (for simplicity, we consider a one dimensional lead, so that k is a scalar). Finally, the dot-lead coupling is

$$H_T = \sum_{k\sigma} t_{k\sigma} c_{k\sigma}^\dagger d_\sigma + t_{k\sigma}^* d_\sigma^\dagger c_{k\sigma}, \tag{9}$$

which desctribes a spin-preserving lead-dot tunneling with, in general complex and spin and energy dependent, tunneling amplitudes $t_{k\sigma}$.

We now repeat the standard calculation [5–9] with minor adjustments to arrive at the inelastic spin decay rather than the co-tunneling current. To this end, we define the transition rate by the Fermi's Golden rule formula

$$\Gamma_{i\to f} = \sum_{i_{\text{lead}}} p_{i_{\text{lead}}} \sum_{f_{\text{lead}}} \frac{2\pi}{\hbar} |\langle f_{\text{dot}} \otimes f_{\text{lead}}|G|i_{\text{dot}} \otimes i_{\text{lead}}\rangle|^2 \delta(E_i - E_f), \tag{10}$$

where i and f are the initial and final states with energies E_i and E_f, respectively, considered to be separable (to the lead and dot components) eigenstates of the unperturbed system described by $H_0 = H_D + H_L$. As we are not conditioning the transitions on the states of the lead, the rate is summed over all possible initial lead states, with the corresponding probabilities $p_{i_{\text{lead}}}$, and all lead final states. The former gives the prescription for a replacement $\sum_{i_{\text{lead}}} p_{i_{\text{lead}}} |i_{\text{lead}}\rangle\langle i_{\text{lead}}| \to \rho_{\text{lead}}^{\text{thermal}}$, with the latter the equilibrium density matrix corresponding to a system with Hamiltonian H_L, at a temperature T. Finally, G is the transition operator which can be expanded in powers of the tunneling term

$$G = H_T + H_T \frac{1}{E - H_0 - i\gamma} H_T + \dots \tag{11}$$

with $E = E_i = E_f$. The two terms describe, respectively, the direct tunneling and the co-tunneling, and γ is a regularization factor [10].

Simple results can be derived in the well justified case of a negligible dependence of the tunneling amplitudes on the wave vector, $t_{k\sigma} \approx t_\sigma$. Using the first term in Eq. (11) gives in this limit the following expression for the direct tunneling rates defined in Eq. (1)

$$\Gamma_\sigma = \frac{2\pi}{\hbar}|t_\sigma|^2 g_F, \tag{12}$$

where we denoted $\Gamma_\sigma \equiv \Gamma_{\sigma\to 0} = \Gamma_{0\to\sigma}$ and g_F is the density of states in the lead at the Fermi energy. Similarly, keeping only the second term in Eq. (11) gives the co-tunneling rate for a spin-flip (from σ to the opposite value $\overline{\sigma}$) of a single electron occupying the dot,

$$\Gamma_{\sigma\to\overline{\sigma}} = \frac{\hbar}{2\pi}\Gamma_\sigma\Gamma_{\overline{\sigma}} \int \mathrm{d}\epsilon f_{\mathrm{FD}}(\epsilon+\sigma E_z)\left[1 - f_{\mathrm{FD}}(\epsilon+\overline{\sigma}E_z)\right] \left|\frac{1}{\epsilon-\mu(2)+i\gamma} - \frac{1}{\epsilon-\mu(1)-i\gamma}\right|^2, \tag{13}$$

where $\mu(2) = \epsilon_S - \epsilon_1$ is (the spin independent part of) the energy cost to add a second electron into the dot. The expression can be further simplified if the dot is deep in the Coulomb blockade, so that the charge excitation energies are much larger than the temperature, namely $\mu(1) \ll \mu_F \ll \mu(2)$ are well fulfilled on the energy scale of the temperature, $k_B T$. The energy dependence of the last term in Eq. (13) can be then neglected, replacing $\epsilon \to \mu_F$, and the remaining integral can be evaluated resulting in

$$\Gamma_{\sigma\to\overline{\sigma}} = \frac{\hbar}{2\pi}\Gamma_\sigma\Gamma_{\overline{\sigma}} \frac{2\sigma E_z}{\exp\left(\frac{2\sigma E_z}{k_B T} - 1\right)} \left(\frac{1}{\mu(2)-\mu_F} + \frac{1}{\mu_F - \mu(1)}\right)^2, \tag{14}$$

where we also neglected the regularization factors. In the large temperature limit, $k_B T \gg E_z$, the temperature dependent factor becomes $k_B T$, while in the opposite limit, $k_B T \ll E_z$, it gives $2E_z$ for $\sigma =\downarrow$, and 0 for $\sigma =\uparrow$. However, Eq. (14) is already in the form which was used to fit the data and is thus the final result of this section.

---

* tomohiro.otsuka@riken.jp

† tarucha@ap.t.u-tokyo.ac.jp

[1] S. Amasha, K. MacLean, Iuliana P. Radu, D. M. Zumbül, M. A. Kastner, M. P. Hanson, and A. C. Gossard, *Spin-dependent tunneling of single electrons into an empty quantum dot*, Phys. Rev. B **78**, 041306(R) (2008).

[2] P. Stano and Ph. Jacquod, *Spin-dependent tunneling into an empty lateral quantum dot*, Phys. Rev. B **82**, 125309 (2010).

[3] M. Yamagishi, N. Watase, M. Hashisaka, K. Muraki, and T. Fujisawa, *Spin-dependent tunneling rates for electrostatically defined GaAs quantum dots*, Phys. Rev. B **90**, 035306 (2014).

[4] T. Otsuka, T. Nakajima, M. R. Delbecq, S. Amaha, J. Yoneda, K. Takeda, G. Allison, T. Ito, R. Sugawara, A. Noiri, A. Ludwig, A. D. Wieck and S. Tarucha, *Single-electron Spin Resonance in a Quadruple Quantum Dot*, Sci. Rep. **6**, 31820 (2016).

[5] D. V. Averin and Yu. V. Nazarov, *Virtual electron diffusion during quantum tunneling of the electric charge*, Phys. Rev. Lett. **65**, 2446 (1990).

[6] M. R. Wegewijs, and Yu. V. Nazarov, *Inelastic co-tunneling through an excited state of a quantum dot*, arXiv:cond-mat/0103579.

[7] V. N. Golovach and D. Loss, *Transport through a double quantum dot in the sequential tunneling and cotunneling regimes*, Phys. Rev. B **69**, 245327 (2004).

[8] J. Lehmann and D. Loss, *Cotunneling current through quantum dots with phonon-assisted spin-flip processes*, Phys. Rev. B 73, 045328 (2006)

[9] P. Stano, J. Klinovaja, F. R. Braakman, L. M. K. Vandersypen, and D. Loss, *Fast Long-Distance Control of Spin Qubits by Photon Assisted Cotunneling*, Phys. Rev. B 92, 075302 (2015).

[10] G. Begemann, S. Koller, M. Grifoni, and J. Paaske, *Inelastic cotunneling in quantum dots and molecules with weakly broken degeneracies*, Phys. Rev. B **82**, 045316 (2010).
